# Factors associated with the prevalence of refractive errors among medical students in the United Arab Emirates

**DOI:** 10.12688/f1000research.170570.1

**Published:** 2025-09-22

**Authors:** Mansour A. Alsaadi, Mansour S. Aouda, Maha D. Almheiri, Ghaith A. Al Amri, Sara H. Ouda, Ahmed M. Alsaadi, Asem Alqudah, Abdelwahab Aleshawi, Mohammed Z. Allouh

**Affiliations:** 1 United Arab Emirates University College of Medicine and Health Sciences, Al Ain, Abu Dhabi, 15551, United Arab Emirates; 2 Department of Ophthalmology, Zayed Military Hospital, Abu Dhabi, Abu Dhabi, United Arab Emirates; 3 Department of Ophthalmology, Sheikh Khalifa Medical City, Abu Dhabi, Abu Dhabi, United Arab Emirates; 4 Department of Special Surgery, Jordan University of Science and Technology Faculty of Medicine, Irbid, Irbid Governorate, 22110, Jordan

**Keywords:** Hyperopia, medical students, myopia, refractive errors

## Abstract

**Background:**

Refractive errors (REs) remain a leading cause of visual impairment worldwide. Medical students frequently report a high burden of REs, possibly attributable to genetic influences and lifestyle factors like extended near-work activities and limited outdoor exposure. Limited data exist on the prevalence and determinants of REs among medical students in the United Arab Emirates (UAE). This study aimed to determine the prevalence of REs in this population and to identify the key potential risk factors associated with them.

**Methods:**

A cross-sectional survey was conducted among medical students at the United Arab Emirates University. A validated 14-item questionnaire assessed the presence and type of REs, demographic and physical characteristics, educational level, family history, electronic device usage, and sunlight exposure. The survey was disseminated electronically between November 2023 and February 2024. Proper statistical analysis was employed to determine key predictors of REs.

**Results:**

Among 328 participants, the overall prevalence of refractive errors was 61.3%, with myopia (with or without astigmatism) accounting for 84.1% of these cases. Female students exhibited more than three-fold higher risk of having REs than males (p = 0.01), and students with a first-degree relative affected had a 7.6-fold greater likelihood of having REs (p < 0.001). Most students with REs (77.1%) used spectacles for correction, and only 11 had laser refractive surgery. Finally, body weight significantly modulated RE type, favoring hyperopia among heavier participants (p < 0.05).

**Conclusion:**

This study reveals a high prevalence of REs among medical students in UAE, with myopia being the predominant type. The key risk factors associated with this high prevalence were female sex and positive family history. These findings underscore the need for targeted preventative and corrective strategies.

## Introduction

Refractive errors (REs) are a major public health problem worldwide and are considered the most common cause of visual impairment. ^1, 2^ Previous studies have investigated the prevalence of REs and their associated risk factors in different countries and populations. A wide range of prevalence rates (20–80%) has been reported. ^3– 6^ Various factors were associated with an increased risk of REs among different populations. The most common risk factors discovered were increasing educational levels, higher individual income, professional occupation, better housing, reduced sunlight exposure, and positive family history. ^7– 12^ Moreover, the types of REs were found to have different prevalence rates in different age groups, with myopia being relatively common in the 10–29-year age group, hyperopia having an increasing prevalence after 40 years, and astigmatism having a decreasing trend with ages from 6 to 90 years. ^13, 14^

The prevalence of REs among medical students has been investigated in many countries. In Singapore, the prevalence of myopia among medical students was 89.8%. ^15^ Nigerian medical students had a prevalence of 79.5%, and Turkish medical students had a prevalence of myopia of about 32.9%. ^16, 17^ In Europe, the European Eye Epidemiology (E3) Consortium concluded that the prevalence of REs in 2015 was distributed as follows: 30.6% for myopia, 25.2% for hyperopia, and 23.9% for astigmatism. ^18^ In Jordan, a 2023 study reported that three-quarters of medical students suffered from REs (75% prevalence). ^19^ Among these students, 82.6% had myopia or myopia with astigmatism. ^19^

To the best of our knowledge, no study has investigated the prevalence of REs and their associated risk factors among medical students in the United Arab Emirates (UAE). This lack of research underscores the objective of our work. This study aimed to assess the prevalence of different types of REs among medical students in the UAE and elucidate the possible risk factors associated with these errors.

## Methods

### Study design and setting

This was a cross-sectional study performed through a survey that involved medical students at the United Arab Emirates University (UAEU), the largest university in the UAE with the only public medical school in the country. According to the 2023/2024 academic year record, the total number of medical students at the UAEU was 602. Ethical approval for the study protocol was obtained from the Research Ethics Committee at the UAEU (approval # ERSC_2023_3755). The committee waived the need for written or verbal consent because the study was based on an anonymous questionnaire, filled out voluntarily and without any personally identifiable information. The questionnaire aimed to assess the incidence of different types of REs (myopia, hyperopia, and astigmatism) among medical students and their associated risk factors.

### Questionnaire design

The survey was based on a questionnaire developed by our Jordanian collaborators, ^19^ with minor modifications to reflect the study curriculum at the UAEU and certain physical aspects of the students that might be associated with REs (height and weight). The survey questions were reviewed by several experts, including ophthalmologists and statisticians, who deemed the questions to be reasonable. The questionnaire was developed in English.

The complete questionnaire comprised 14 systematic, accurate questions. Eight questions inquired about the risk factors associated with REs. Four of these were related to the student’s physical characteristics: sex, age, height, and weight. The other four were related to the student environment: study level, family history, use of electronic devices, and exposure to sunlight.

Notably, the medical program at the UAEU is divided into three phases. These are the premedical phase, covering the foundation and basic medical sciences; the preclinical phase, where medical students learn integrated medical sciences in systemic modules; and the clinical phase, where medical students receive proper clinical training through hospital rotations. One of the objectives of this study was to verify if there was an association between the study level (phase) and the development of REs among medical students. Therefore, the frequency of REs was compared among the students in the three different medical phases.

One question related to the family history of REs inquired about having a first-degree relative (parent or sibling) with REs. Additionally, two questions related to the student’s behavior inquired about the daily period of using electronic devices and the daily period of sunlight exposure, as it has been established that myopia is significantly associated with reduced sunlight exposure time. ^11^

The remaining six questions were related to the: presence of an RE, type of RE, dioptric degree, diagnosis age, correction method used, and laser refractive surgery. The REs were classified into five categories: myopia alone, myopia and astigmatism, hyperopia alone, hyperopia and astigmatism, and astigmatism alone. The last question inquired about why the student did not opt to undergo laser refractive surgery because we also aimed to investigate the students’ attitudes toward this ultimate correction method.

### Sampling

A link to the survey, which was developed through Google Forms, was distributed to the medical students at the UAEU through repeated official emails between November 2023 and February 2024. This data collection method was more convenient for the students and researchers, and it was rapid and efficient. The link included a preface that explained the aim of the study; a statement that ensured the optionality, anonymity, and confidentiality of the study; the right to withdraw anytime; and the consent to participate.

### Statistical analysis

Statistical analyses were performed using IBM SPSS Statistics software (standard version 29.0.0.0, IBM, Armonk, NY, USA). The data were extracted into an Excel file and then transferred to an SPSS spreadsheet. Data were expressed as frequency (percentage) for categorical variables and mean ± standard deviation of the mean (SD) for continuous variables. Cases containing contradictory answers were excluded from the analysis. The level of statistical significance was set at p < 0.05. This was determined using Pearson’s chi-squared test for categorical variables and student’s *t*-test for continuous variables. Multivariate logistic regression was also applied to determine the main predictors of the incidence of REs among medical students.

## Results

### Prevalence of REs

A total of 336 students completed the survey, producing a response rate of 55.8% (336/602). However, eight participants were excluded because they provided contradictory answers, such as answering that they had no REs and had myopia as an RE or that they had no REs and used spectacles as a correction method. Three of these eight cases were excluded because the students answered that they used spectacles as a correction method and also answered that they had undergone laser refractive surgery as a correction method.

Among the 328 participants, there were 201 (61.3%) students with REs and 127 (38.7%) without REs. Regarding sex, there were 239 (72.9%) females and 89 (27.1%) males. Most of the participants were in the age range of 17–25 years. The students who completed the survey were distributed according to the three phases of the medical curriculum at the UAEU as follows: 161 (49.1%) in the premedical phase, 98 (29.9%) in the preclinical phase, and 69 (21.0%) in the clinical phase. Most of the students (282/328, 86.0%) had a first-degree relative with an RE and had exposure to sunlight for less than 3 h per day (275/328, 83.8%). Additionally, most of the students (304/328, 92.7%) used electronic devices for more than 5 h per day.

### Risk factors associated with REs

The independent risk factors that might be associated with the development of REs are summarized in Table 1. Most of these factors were independently associated with the prevalence of REs among medical students in the UAE. Student sex was significantly associated with REs (p < 0.01), with female students having a higher-than-expected prevalence (67.4%). Older and shorter students were more likely associated with REs (p < 0.05). Moreover, the statistical analysis revealed a significant association between the study level and the prevalence of REs (p < 0.05). Students in the premedical phase had a less-than-expected prevalence rate of REs (54.0%), while students in the clinical phase had a more-than-expected prevalence rate (72.5%) ( Table 1). The presence of a first-degree relative with REs was significantly associated with the prevalence of REs (p < 0.001), as most of the students who had a first-degree relative with REs (68.1%) had REs, while most students who did not have a first-degree relative with REs (80.4%) did not have REs. There were no significant associations between the use of electronic devices or the exposure to sunlight and the prevalence of REs among the medical students.

**Table 1.** Risk factors associated with refractive errors among medical students in the UAE.

Risk factor	No REs	REs	p-value
**Sex**			<0.001
Female	78 (32.6%)	161 (67.4%)↑	
Male	49 (55.1%)	40 (44.9%)	
**Age (y)**	19.5 ± 1.7	20.0 ± 1.7	0.008
**Height (cm)**	165.1 ± 8.7	162.5 ± 7.6	0.007
**Weight (kg)**	63.9 ± 14.4	63.3 ± 16.1	NS
**BMI**			NS
Underweight	21 (42.0%)	29 (58.0%)	
Healthy	58 (38.2%)	94 (61.8%)	
Overweight	39 (41.5)	55 (58.5%)	
Obese	8 (29.6%)	19 (70.4%)	
**Medical level**			0.020
Premedical	74 (46.0%)	87 (54.0%)↓	
Preclinical	34 (34.7%)	64 (65.3%)	
Clinical	19 (27.5%)	50 (72.5%)↑	
**Presence of a first-degree relative with an RE**			<0.001
Yes	90 (31.9%)	192 (68.1%)↑	
No	37 (80.4%)↑	9 (19.6%)	
**Use of electronic devices (h/day)**			NS
0–2	2 (100%)	0 (0.0%)	
2–5	12 (54.5%)	10 (45.5%)	
5–8	57 (39.3%)	88 (60.7%)	
> 8	56 (35.2%)	103 (64.8%)	
**Exposure to sunlight (h/day)**			NS
< 3	108 (39.3%)	167 (60.7%)	
3–5	16 (34.0%)	31 (66.0%)	
> 5	3 (50%)	3 (50%)	

Abbreviations: RE, refractive error; NS, not statistically significant (p > 0.05); BMI, body mass index; p, probability; y, year.

Data are presented as mean ± standard deviation for continuous variables and as frequency (percentage) for categorical variables. The p-value was calculated using Pearson’s chi-squared test for categorical variables and the student’s *t*-test for continuous variables. ↑: significantly higher than expected. ↓: significantly lower than expected.

Nevertheless, a multivariate regression analysis model was generated to determine the true predictors of REs from the previously described risk factors. The model revealed that sex and the presence of a first-degree relative with an RE were the only two true predictors of the incidence of REs among medical students in the UAE ( Table 2). Female students had a more than 3-fold greater risk of developing REs than male students (p = 0.01). Additionally, students who had a first-degree relative with an RE had a 7.6-fold higher risk of developing REs than students who did not have one (p < 0.001).

**Table 2.** Multivariate logistic regression model for the predictors of refractive errors among medical students in the UAE.

The predictors	p-value	Odds ratio	95% CI	
			Lower	Upper
**Sex**				
Female	**0.010**	**3.2**	1.316	7.780
Male	Ref	Ref	Ref	Ref
**Age**	0.466	-	0.833	1.489
**Height**	0.708	-	0.953	1.074
**Weight**	0.346	-	0.976	1.071
**BMI**				
Underweight	0.699	-	0.155	16.171
Healthy	0.628	-	0.254	9.691
Overweight	0.930	-	0.280	4.034
Obese	Ref	Ref	Ref	Ref
**Medical Level**				
Premedical	0.709	-	0.224	2.768
Preclinical	0.721	-	0.356	2.045
Clinical	Ref	Ref	Ref	Ref
**Presence of a first-degree relative with an RE**				
Yes	**<0.001**	**7.6**	3.262	17.934
No	Ref	Ref	Ref	Ref
**Use of electronic devices (h/day)**				
0–2	0.999	-	0.000	>1000
2–5	0.182	-	0.178	1.387
5–8	0.309	-	0.453	1.285
>8	Ref	Ref	Ref	Ref
**Exposure to sunlight (h/day)**				
<3	0.506	-	0.284	12.799
3–5	0.315	-	0.374	21.136
>5	Ref	Ref	Ref	Ref

Abbreviations: RE, refractive error; P, probability; CI, confidence interval; BMI, body mass index.

The logistic regression model included the nine variables reported in this table.

### Characteristics of the students with REs and distribution of the different types of REs

Table 3 presents the characteristics of the students with REs and the distribution of the different types of REs among the students. Most of the students with REs were females (80.1%), corroborating the previous finding that sex is a predictor of REs. Additionally, most of the students with REs (95.5%) had a first-degree relative with an RE, which also corroborates the previous results above. Most of the students with REs (83.1%) had exposure to sunlight for less than 3 h/day ( Table 3). However, this is considered a normal situation, taking into consideration the hot environment of the UAE. Similarly, most of the students without REs (108/127, 85.0%) had exposure to sunlight for less than 3 h/day.

**Table 3.** Distribution of the refractive errors among medical students in the UAE.

Studied variable	Frequency	Percentage
**Sex**		
Female	161	80.1%
Male	40	19.9%
**BMI**		
Underweight	29	14.4%
Healthy	94	46.8%
Overweight	55	27.4%
Obese	19	9.5%
Missing cases	4	2.0%
**Medical level**		
Premedical	87	43.3%
Preclinical	64	31.8%
Clinical	50	24.9%
**Presence of a first-degree relative with an RE**		
Yes	192	95.5%
No	9	4.5%
**Use of electronic devices**		
2–5 h	10	5.0%
5–8 h	88	43.8%
>8 h	103	51.2%
**Exposure to sunlight**		
<3 h	167	83.1%
3–5 h	31	15.4%
>5 h	3	1.5%
**Type of RE**		
Myopia alone	99	49.3%
Myopia with astigmatism	70	34.8%
Hyperopia alone	11	5.5%
Hyperopia with astigmatism	13	6.5%
Astigmatism alone	8	4.0%
**Degree of myopia or hyperopia**		
<1	36	17.9%
1–3	90	44.8%
3–6	54	26.9%
>6	11	5.5%
Missing cases	10	5.0%
**Diagnosis age of RE (years)**		
0–5	9	4.5%
5–10	45	22.4%
10–15	72	35.8%
15–20	64	31.8%
>20	8	4.0%
Missing cases	3	1.5%
**Correction method**		
Glasses	155	77.1%
Contact lenses	28	13.9%
Laser surgery	11	5.5%
No corrections	7	3.5%
**Reasons for not performing laser surgery**		
Cost	6	3.0%
Not a candidate	26	12.9%
Prefer other methods	29	14.4%
Thinking later	127	63.2%
I did perform a laser surgery	10	5.0%
Missing cases *	3	1.5%

Abbreviations: BMI, body mass index; RE, refractive error.

* One of the missing cases was of a student who answered that he underwent laser refractive surgery as a correction method in the previous question.

Regarding the types of REs, myopia, with or without astigmatism, was the most frequent type among the medical students with REs (84.1%). This constitutes a myopia prevalence rate of 51.5% of the total sample (169/328). Twenty-four (12.0%) students had hyperopia with or without astigmatism. Only eight (4.0%) students had astigmatism alone, as most astigmatism cases (83 students, 41.3%) were associated with either myopia or hyperopia. Most students with REs (44.8%) had myopia or hyperopia within the range of 1–3 diopters, 26.9% had >3–6 diopters, 17.9% had <1 diopter, and 5.5% had >6 diopters. Regarding the age at diagnosis, approximately two-thirds of the students (67.6%) were diagnosed between 10 and 20 years. Only 4.5% of the students were diagnosed before the age of 5 years; 22.4%, between 5 and 10 years; 35.8%, between 10 and 15 years; 31.8%, between 15 and 20 years; and only 4.0%, above 20 years.

Most of the students with REs (155/201, 77.1%) used eyeglasses for correction. Out of the 201 medical students who had REs, only seven (3.5%) did not use any correction method. Eleven (5.5%) students underwent laser refractive surgery. A question was asked about why the students chose not to perform laser refractive surgery, and most of them (127/201, 63.2%) stated that they were thinking of doing it later. However, 29 students (14.4%) preferred other methods, while the other 26 (12.9%) were not candidates for laser refractive surgery.

### Myopia versus hyperopia

The factors that might be associated with the type of REs (myopia or hyperopia) among medical students in the UAE are summarized in Table 4. Only the students’ heights and weights were significantly associated with the type of REs (p < 0.05). Taller students were associated with hyperopia, while shorter ones were more associated with myopia. Additionally, students with a greater body weight were associated with a higher prevalence of hyperopia, while those with a lower body weight were associated with a higher prevalence of myopia. However, the multivariate regression analysis, including all factors reported in Table 4, revealed that weight was the only predictor of the type of RE. Students with a higher body weight had a 1.175-fold higher prevalence of hyperopia than those with a lower body weight (p < 0.05). None of the remaining factors were significantly associated with the type of RE.

**Table 4.** Risk factors associated with myopia versus hyperopia among medical students in the UAE.

Risk factor	Myopia	Hyperopia	p-value
**Sex**			NS
Female	137 (89.5%)	16 (10.5%)	
Male	32 (80.0%)	8 (20.0%)	
**Age (y)**	20.0 ± 1.7	19.7 ± 1.9	NS
**Height (cm)**	162.0 ± 7.7	166.2 ± 6.9	**0.012**
**Weight (kg)**	62.3 ± 16.6	69.3 ± 13.1	**0.049**
**BMI**			NS
Underweight	28 (96.6%)	1 (3.4%)	
Healthy	77 (86.5%)	12 (13.5%)	
Overweight	43 (81.1%)	10 (18.9%)	
Obese	17 (94.4%)	1 (5.6%)	
**Medical level**			NS
Premedical	69 (83.1%)	14 (16.9%)	
Preclinical	59 (95.2%)	3 (4.8%)	
Clinical	41 (85.4%)	7 (14.6%)	
**Presence of a first-relative with an RE**			NS
Yes	163 (88.6%)	21 (11.4%)	
No	6 (66.7%)	3 (33.3%)	
**Use of electronic devices (h/day)**			NS
0–2	0 (0%)	0 (0%)	
2–5	7 (70.0%)	3 (30.0%)	
5–8	73 (86.9%)	11 (13.1%)	
>8	89 (89.9%)	10 (10.1%)	
**Exposure to sunlight (h/day)**			NS
<3	143 (88.8%)	18 (11.2%)	
3–5	24 (82.8%)	5 (17.2%)	
>5	2 (66.7%)	1 (33.3%)	

Abbreviations: RE, refractive error; BMI, body mass index; NS, not statistically significant (p > 0.05); p, probability; y, year.

Data are presented as mean ± standard deviation for continuous variables and as frequency (percentage) for categorical variables. The p-value was calculated using Pearson’s chi-squared test for categorical variables and the student’s *t*-test for continuous variables.

## Discussion

To our knowledge, this is the first study to investigate the prevalence of REs among medical students in the UAE. We found that sex and the presence of a first-degree relative with an RE were the main risk factors associated with the prevalence of REs among these medical students. Female medical students had a higher prevalence of REs than male medical students. Almost all students with REs (95.5%) had a first-degree relative with an RE. The physical characteristics, among the presence of other confounding factors, did not show any association with the presence of REs. However, when tested for the type of RE, weight was significantly associated with the type of RE, as students with a greater body weight tended to have a higher prevalence of hyperopia.

The higher prevalence of REs among female students can be explained by the fact that most of the participants were females (~73%). This can be explained by the higher number of females than males who were admitted to the college: 488 females compared to 197 males. Similarly, a study on the prevalence of REs in Jordan revealed that 63% of the participants were females. ^19^ Additionally, the prevalence of REs was significantly higher among female school students compared to that among male students in Egypt. ^12^ However, the prevalence rate of myopia did not show a significant difference between female and male Turkish medical students. ^16^ A study in Singapore also revealed no difference in the prevalence rate of myopia between female and male medical students. ^15^ These findings collectively indicate that the influence of sex on the prevalence of REs may be associated with the ethnicity of the studied population.

Additionally, the prevalence of REs among medical students in the UAE was significantly higher with the presence of a first-degree relative who has an RE. The results showed that 95.5% of medical students who had REs also had a first-degree relative with an RE. A previous study from Egypt showed that the prevalence of REs was significantly higher among school children with a positive family history of REs compared to that in those with no family history; however, consanguinity did not affect the prevalence of REs. ^12^ Two multicenter, longitudinal studies revealed that the risk of being myopic was increased for children with two versus one or no parents with myopia. ^20, 21^ In summary, having a first-degree relative with an RE increases the chance of developing an RE; however, how REs are inherited is not completely understood. ^22^

There is inconsistency in the literature regarding the association of physical characteristics, including height and weight, with the prevalence of different types of REs. Several previous studies suggested a positive association between height and myopia. Joseph et al. ^23^ revealed that increased height was a risk factor for myopia in the southern Indian adult population. In contrast, Hashemi et al. ^24^ found no correlation between height and myopia among Iranian university students, indicating that the relationship between height and REs may vary by ethnicity or age. A gender-specific pattern was noted by Machluf et al., ^25^ where shorter males exhibited a higher prevalence of bilateral myopia, while no significant relationship was observed in females. In the context of stunting, Sharma et al. ^26^ found that neither stunting nor height was associated with REs after adjusting for confounding factors such as age and parental education among rural Chinese schoolchildren.

Also, the impact of weight on REs is variable, with some studies indicating a protective effect against myopia at higher weight indices. For example, consistent with our findings, Saw et al. ^27^ observed that children with greater body weights in Singapore were more hyperopic, suggesting that the relationship between weight and REs may differ across populations and involve other factors such as lifestyle and nutrition. Moreover, Shi et al. ^28^ reported an inverse correlation between the Weight-Adjusted Waist Index (WWI) and myopia severity, indicating that a higher WWI could be protective against severe myopia. In contrast, Hashemi et al. ^24^ observed a direct correlation between increased weight and higher odds of myopia among Iranian students, highlighting weight as a broader risk factor for REs. The relationship between physical characteristics (height and weight) and REs appears to be multifactorial, shaped by a complex interplay among genetic, environmental, nutritional, and lifestyle determinants.

A limitation of this study could be the possibility of selection bias, as students with REs might be more likely to participate. Also, the response rate of 55.8% might be considered inadequate by some researchers. However, there is a general consensus that at least half the survey cohort must complete the survey to be acceptable. ^29^ To avoid these limitations, many reminder emails were sent to the students to encourage participation regardless of the presence or absence of REs. Additionally, the survey period was extended for four months to recruit as many participants as possible.

## Conclusion

The overall prevalence rate of REs among medical students in the UAE was high (61.3%). Myopia, with or without astigmatism, constituted the most frequent type of RE in these students, with a prevalence rate of 51.5% of the total sample (169/328). Moreover, the two main risk factors associated with this prevalence were sex and the presence of a familial history. Female medical students had a higher prevalence of REs than male students. Almost all students with REs (95.5%) had a first-degree relative with an RE.

The physical characteristics, among the presence of other confounding factors, did not show strong associations with the presence of REs. However, when tested for the type of RE, higher body weights were associated with a higher tendency toward hyperopia than myopia. However, the association between physical characteristics and REs is complex and likely influenced by genetic, environmental, and lifestyle factors. Further research is needed to expand this survey to a national level in the UAE to develop targeted prevention strategies for the population.

## Ethics and consent

Ethical approval for the study protocol was obtained from the Research Ethics Committee at the UAEU (approval # ERSC_2023_3755). The committee waived the need for written or verbal consent because the study was based on an anonymous questionnaire, filled out voluntarily and without any personally identifiable information.

## Grants information

This research was supported by grants from the United Arab Emirates University to M. Z. Allouh; a CMHS grant (Grant code: G00004697, Fund number: 12M176), and a SURE Plus grant (Grant code: G00005273, Fund number: 12M280).

## Data Availability

Figshare: Prevalence of Refractive Errors among Medical Students in United Arab Emirates, https://doi.org/10.6084/m9.figshare.30093157. ^30^ This project contains the following underlying data:
1. Original Dataset Data are available under the terms of the Creative Commons Attribution 4.0 International license (CC-BY 4.0).
