# Supplementary material for: Factors associated with the prevalence of refractive errors among medical students in the United Arab Emirates
Source: F1000Res. 2025 Sep 22;14:954. [Version 1] doi: 10.12688/f1000research.170570.1 (PMC12559844; doi:10.12688/f1000research.170570.1)
Supplement: Peer review history

# Reviewer report: Reviewer response for version 1

**DOI:** 10.5256/f1000research.188041.r420436

**Published:** 2025-10-27

**Authors:** Saranya Sachi Balasubramaniam

**Affiliations:** 1 Acchutha Institute of Optometry, Tamil Nadu, India

There is no mention regarding the ethnicity of the participants. Please look into the ethnicity. Asian, South East Asian populations have more myopia, compared to the rest of the world. 

 The selection bias: the participants are usually those who are proactive students, probably those who tend to do more reading and close work too. Consider this factor too. 

 Otherwise the article looks good, with relevant points and appropriate analysis.

Is the work clearly and accurately presented and does it cite the current literature?

Yes

If applicable, is the statistical analysis and its interpretation appropriate?

Yes

Are all the source data underlying the results available to ensure full reproducibility?

Yes

Is the study design appropriate and is the work technically sound?

Yes

Are the conclusions drawn adequately supported by the results?

Yes

Are sufficient details of methods and analysis provided to allow replication by others?

Yes

Reviewer Expertise:

Optometry, Employability Skills for Optometry Graduates,

I confirm that I have read this submission and believe that I have an appropriate level of expertise to confirm that it is of an acceptable scientific standard.

# Reviewer report: Reviewer response for version 1

**DOI:** 10.5256/f1000research.188041.r420432

**Published:** 2025-10-07

**Authors:** Hassan A Aljaberi

**Affiliations:** 1 Al-Mustaqbal University, Hillah, Iraq

Overall summary

 This study examines the prevalence and correlates of refractive errors (REs) among medical students at the United Arab Emirates University. The topic is important for public health and ophthalmic practice in the Gulf region, addressing a local evidence gap. The manuscript is generally well-written and provides useful epidemiological data. However, several methodological and analytical aspects require clarification or extension to strengthen the validity and interpretability of the findings.

 Major comments

**1. Rationale and positioning: **The Introduction should more clearly justify the focus on medical students in the UAE and articulate what is unique about this population or context (e.g., educational intensity, digital exposure, climate/outdoor time). Add comparison to related UAE/GCC student populations (non‑medical) to motivate the study’s contribution.

**2. Study design, sampling, and potential biases: **Because the study is cross‑sectional and based on a self‑administered online survey, please discuss and, where possible, assess selection and recall biases. Consider comparing early versus late responders to evaluate response bias and provide any available information on representativeness relative to the full medical student cohort (sex, phase).

**3. Outcome definition and modeling strategy: **The binary outcome combining all REs (myopia, hyperopia, astigmatism) obscures heterogeneity. Given that categories are not mutually exclusive, consider using multinomial/ordinal models, or separate models for each RE subtype; at a minimum, justify the chosen approach and discuss its implications.

**4. Model specification and diagnostics: **Report full model specification with all covariates; provide adjusted odds ratios with 95% CIs in the main text or tables. Assess multicollinearity (e.g., variance inflation factors) and model fit/calibration. Clarify the variable selection process (theory-driven vs. data-driven) and the rationale for including height/weight alongside BMI.

**5. Use of age at diagnosis: **Age at diagnosis is a strength of the dataset. Consider survival/duration analysis (time‑to‑RE onset) or at least stratified analyses by age‑of‑onset bands to provide additional insight.

**6. Interpretation of sex differences: **Avoid attributing the higher RE prevalence in females to a larger female subsample; prevalence is a proportion and not driven by subsample size if sampling is representative. Discuss alternative explanations (behavioral, educational load, near‑work patterns) and consider sensitivity analyses.

**7. Height/weight associations with RE subtype: **Provide physiological or behavioral hypotheses (e.g., body growth, ocular axial length, hormonal factors) to contextualize the observed associations. Indicate whether findings persist after adjusting for potential confounders (e.g., study phase, device use, family history).

**8. Limitations: **Expand the limitations to include reliance on self‑reported RE status (lack of clinical confirmation), potential misclassification, single‑institution sampling (generalizability), and inability to infer causality from cross‑sectional data.

 Minor comments

 • Abstract: replace vague phrasing (e.g., “proper statistical analysis”) with specific methods (chi‑square tests; t‑tests; multivariable logistic regression).

 • Keywords: add “United Arab Emirates” and include all RE subtypes (myopia, hyperopia, astigmatism).

 • Introduction: at first mention of REs, explicitly list the three subtypes for clarity.

 • Tables: Table 1 and Table 3 contain overlapping information; consider combining to reduce redundancy and add clear footnotes on statistical tests.

 • Figures: include at least one visualization (e.g., distribution of RE subtypes; prevalence by sex/phase).

 • Methods: specify questionnaire validation details (source, any psychometrics such as Cronbach’s alpha) and whether RE status was self‑reported or clinically confirmed.

 • Statistical analysis: state how missing data were handled; confirm assumptions for tests; report exact p‑values when appropriate.

 • Results: present adjusted ORs with 95% CIs directly in the text or a primary table; ensure consistent decimal places and units.

 • Discussion: expand comparative discussion versus regional/international studies and clarify potential reverse causality for device‑use variables.

 • Data availability: briefly describe dataset structure (variables, coding) to aid reuse; keep the Figshare link as provided.

 • Writing/style: reduce repetition between Introduction and Discussion; use past tense for results; ensure consistent use of abbreviations (REs).

 Suggested text edits (optional)

 Abstract – Methods: “Statistical analyses included chi‑square tests, t‑tests, and multivariable logistic regression to identify predictors of refractive errors.”

 Keywords: “United Arab Emirates; refractive errors; myopia; hyperopia; astigmatism; medical students.”

 Limitations: “This cross‑sectional, self‑reported survey from a single institution may be subject to selection and recall biases; lack of clinical confirmation may lead to outcome misclassification; generalizability beyond UAEU medical students is limited.”

 Mandatory reviewer questions (to be copied into the online form)

**Competing interests: **I declare that I have no competing interests.

**Methodological soundness: **Partly. The study design is suitable for prevalence estimation; however, the modeling strategy and bias assessment require further clarification.

**Statistical analysis: **Partly appropriate; recommend multinomial modeling or subtype-specific analyses, along with diagnostics for multicollinearity and model fit.

**Data availability/reproducibility: **Yes. Data are available; recommend adding a brief data dictionary/variable description.

**Ethics: **Ethical approval has been obtained, and the design includes appropriate anonymity and consent waivers.

 Recommendation

 Approved with reservations (pending clarifications to methodology and expanded analyses as noted above).

Is the work clearly and accurately presented and does it cite the current literature?

Partly

If applicable, is the statistical analysis and its interpretation appropriate?

Partly

Are all the source data underlying the results available to ensure full reproducibility?

Yes

Is the study design appropriate and is the work technically sound?

Partly

Are the conclusions drawn adequately supported by the results?

Partly

Are sufficient details of methods and analysis provided to allow replication by others?

Partly

Reviewer Expertise:

Optometry, Ophthalmology, Visual Sciences, Refractive Surgery, Eye Health, Medical Device Engineering

I confirm that I have read this submission and believe that I have an appropriate level of expertise to confirm that it is of an acceptable scientific standard, however I have significant reservations, as outlined above.

## References

[1] : Prevalence of refractive errors among school students in Iraq — A systematic review and meta-analysis. *Journal of Optometry* .2025;18(1) : 10.1016/j.optom.2024.100534 10.1016/j.optom.2024.100534 PMC1192508340037119

# Reviewer report: Reviewer response for version 1

**DOI:** 10.5256/f1000research.188041.r417089

**Published:** 2025-10-06

**Authors:** Qihui Chen

**Affiliations:** 1 China Agricultural University, Beijing, China

Reviewer report: Factors associated with the prevalence of refractive errors among medical students in the United Arab Emirates

 General comments

 The paper under review examines factors associated with the prevalence of refractive errors among medical students in the United Arab Emirates (UAE) (all attending the UAE university) using a cross-sectional survey dataset. The study has some original features (such as being the first to look at medical students in the UAE), and the text is, in general, well-written; however, the statistical analysis could have been done in a more in-depth way to detect more granular patterns between the factors examined and the prevalence of refractive errors (REs), as discussed in my comments below. My more specific comments are divided into “major problems” and “minor issues”.

 Major problems

1. Motivation. The Introduction should explain clearly why the study focuses on medical students in the UAE. Is there anything particular about medical students or about the UAE that motivates the study? What new knowledge can we learn from a study focusing on medical students in the UAE?
2. Related to my previous point, the “literature review” section in the Introduction should include studies on the prevalence of REs among other types of students in the UAE to provide some background information.
3. As acknowledged in the “limitations” part, one major concern is the potential selection bias generated by the participants’ voluntary participation—those who were more interested in the study or those suffering more from REs were more likely to participate. The authors did not address this issue effectively. However, given that repeated emails were sent to the students, the authors can test whether selection bias is indeed a concern by examining whether those who agreed to participate immediately after the first email was sent and those who agreed after multiple emails were sent. Alternatively, the authors could include the number of emails sent (or the time elapsed) before a student accepted the invitation as an additional control variable in the regression models to see if it makes a difference.
4. As mentioned in the text, REs examined in the study include myopia, hyperopia, and astigmatism, which are neither mutually exclusive nor independent (see Table 3, “type of RE”). Thus, a Multinomial logit model is a better modelling device than separate logit models. In any case, pooling three REs together to define the outcome measure seems unreasonable.
5. The dataset used contains unique information on the age at which REs were developed. This provides an opportunity to fit duration/hazard models. I suggest that the authors try this direction.
6. The choice of risk factors is not clearly explained. Why these risk factors? The authors should provide both theoretical reasons and empirical evidence from previous studies to support their choice. For example, a recent paper in China found that out-of-school private tutoring was a factor contributing to myopia among middle school students (Chen et al., 2024).
7. Related to my previous points, one reason why the significance of many factors disappeared in the multivariable regression model is the presence of multicollinearity among some of these factors. Thus, the authors should provide a clear rationale behind the choice of risk factors and perform a correlation analysis before running a regression to avoid putting many highly correlated factors in the model.
8. Interpretations of the findings should be strengthened. First, the interpretation of previous findings in the Introduction (page 3, 1 ^st^ paragraph) seems misleading. For example, while higher income has been found to be positively correlated with a higher prevalence of REs, calling higher income a risk factor seems unreasonable—do you suggest that people should give away some of their income to protect their eyes? Income itself won’t cause vision problems. It is something else related to income (e.g., being able to afford a big TV screen or more video games) that causes the problems. Second, some interpretations of the findings of this paper are incorrect. In particular, the authors attributed the gender difference in RE prevalence to the larger number of female participants in the sample. But “prevalence” is a “proportion” measure. If both male and female samples are representative of the UAE University medical student population, the difference in subsample size should not matter. (If it matters, then what about having a first-degree relative with REs?—Your data includes more participants having a first-degree relative with REs than those without; is its bigger sample size driving its significant effect?) An alternative explanation is that female students work harder than male students, which seems more natural. If you have test scores data, this can be easily verified. Third, I suggest that authors further explore their data to aid in interpreting the findings. For example, the authors reviewed some activities (e.g., the use of electronic devices) as risk factors for RE prevalence (Table 4). However, these activities could be the result of REs (e.g., Huang and Chen, 2023). Using them to explain RE prevalence could lead to a reverse causality problem. Finally, the authors did not explain why having a first-degree relative with REs predicts one’s RE prevalence. The authors mentioned “how REs are inherited is not completely understood,” but inheritance may not be the only explanation. If the whole family studied hard when young, then all members could have developed REs, regardless of what their genes look like. In that case, it is family tradition that drives the first-degree relative–REs relationship.

Minor issues

1. Abstract: In the “methods” part, the author mentions “proper statistical analysis.” I suggest that the author replace “proper” with a more specific term, such as the names of the models used (e.g., logit models).
2. Keywords: Consider adding “United Arab Emirates.” Also, why only include “myopia” but not these other REs? 
3. Introduction. Should mention myopia, hyperopia, and astigmatism the first time you mention REs (i.e., page 3, 1 ^st^ sentence). 
4. The Introduction includes a paragraph discussing previous studies (page 3, 1st paragraph), but that section is by no means comprehensive. I would suggest that the author add a more detailed literature review section to better position their own study.
5. Much of the content in Table 3 is redundant, as Table 1, column 2, contains the same information. I suggest that the author combine the two tables.

References

1. refer to 1, 2

Is the work clearly and accurately presented and does it cite the current literature?

Yes

If applicable, is the statistical analysis and its interpretation appropriate?

Partly

Are all the source data underlying the results available to ensure full reproducibility?

Yes

Is the study design appropriate and is the work technically sound?

Partly

Are the conclusions drawn adequately supported by the results?

Partly

Are sufficient details of methods and analysis provided to allow replication by others?

Yes

Reviewer Expertise:

Health Economics

I confirm that I have read this submission and believe that I have an appropriate level of expertise to confirm that it is of an acceptable scientific standard, however I have significant reservations, as outlined above.

## References

[1] Chen, Q., Huang, J., Pei, C. 2024. A neglected health burden of shadow education?—Effects of private supplementary tutoring on middle school students’ vision in China. Children and Youth Services Review, 148, 107463.

[2] Huang, J., Cai, Y., Chen, Q. 2024. Will disease awareness induce healthier behavior?―A regression-discontinuity analysis of effects of myopia diagnosis among Chinese adolescents. Applied Economics 56(59),8991-9013.
